# A compact uniform circular array antenna for circularly polarized higher-order OAM beam generation through modes superposition

**DOI:** 10.1371/journal.pone.0337197

**Published:** 2026-01-30

**Authors:** Umar Fayyaz, Yosef T. Aladadi, Abdul Aziz, Shahab Ahmad Niazi, Syed Tamoor Shah, Sulaiman Al-Sowayan, Zaid Ahmed Shamsan, Rifaqat Hussain

**Affiliations:** 1 Faculty of Engineering and Technology, The Islamia University of Bahawalpur, Punjab, Pakistan; 2 Department of Electrical Engineering, College of Engineering, Imam Mohammad Ibn Saud Islamic University (IMSIU), Riyadh, Saudi Arabia; 3 National University of Science and Technology NUST H-12, Islamabad, Pakistan; 4 School of Electronic Engineering and Computer Science, Queen Mary University of London, London, United Kingdom; Manipal Academy of Higher Education, INDIA

## Abstract

This paper presents the design and validation of a compact uniform circular array (UCA) antenna for the generation of a higher-order orbital angular momentum (OAM) beam. The array combines the fifth-order OAM generated by individual ring patch antenna elements with the first-order OAM mode of the array to radiate the resultant sixth-order OAM beam through mode superposition. A dual-feed configuration is employed to achieve a 90-degree phase difference using differential feed lengths. Sequential rotation of elements ensures circular polarization without the need for complex feeding networks. Characteristic Mode Analysis (CMA) confirms the excitation of *TM*_61_ degenerate modes from ring patch antenna responsible for the fifth-order OAM radiation. The antenna operates at 3.55 GHz and achieves significant mode purity with an axial ratio below 3 dB. The proposed design is a single-layer, low-profile antenna that is suitable for narrowband OAM-based wireless sensor and communication systems, enabling high-order mode generation with a reduced number of array elements.

## Introduction

The increase in demand of data rate has led to the development of communication systems in the last few years. The multiplexing of multiple orthogonal modes in orbital angular momentum (OAM) has been proposed as a promising technique for this demand by researchers [1]. The OAM, an important physical aspect of electromagnetic waves, offers a new degree of freedom for information modulation. In theory, the OAM can have infinite modes that are orthogonal and travel independently in space. Since its application in low-frequency radio in 2007 [2], OAM has been utilized to improve wireless communication capacity and efficiency in microwave and millimeter wave bands [3,4]. Later on in 2010, researchers introduced that a uniform circular array (UCA) can produce a radio beam with OAM properties that are similar to those of a Laguerre-Gaussian beam [5], and confirmed these findings through experiments. Several techniques have been proposed to realize OAM beams in the radio frequency (RF) domain such as single patch antennas [6–8], spiral phase plates[9], uniform circular arrays [10,11] and metasurfaces [12–14]. Among them, uniform circular arrays (UCAs) are effective for generating OAM beams with high mode purity and controllability.

The radial circular array proposed in [15] achieves a dual OAM mode communication system with mode orders ±1 and effective isolation between the channels. However, the approach involves higher complexity due to its multi-layered configuration and lack of circular polarization. Moreover, the design technique may not be easily scalable for higher-order OAM beams, which could require more complex configurations. Although a circularly polarized dual OAM mode antenna is proposed in [16], utilizing triangular patches and a two-loop arrangement of elements along concentric circles, the approach requires a significant number of elements to realize higher-order OAM beams, increasing the overall size and complexity of the system. The design proposed in [17] introduces a planar structure with a concentric arrangement of linearly polarized antenna elements for dual-mode generation. This approach, however, requires different considerations in terms of antenna array configuration and phase control to excite circularly polarized higher-order OAM beams. In [18], an OAM patch antenna and its array are proposed for the generation of fourth OAM wave, based on characteristic mode analysis for optimization. However, the design employs a complex feeding mechanism and a multilayered structure, which adds further complexity in supporting higher-order OAM modes.

A quad-mode UCA is proposed in [19] for the generation of four OAM modes (±1 and ±2) using two concentric arrangements of antenna elements. The proposed antenna, however, requires an individual feeding scheme for each concentric arrangement of elements and a multilayered configuration, thereby increasing the overall size and complexity. Furthermore, the number of required antenna elements increases linearly with the order of the OAM mode. An OAM-based multiplexing system is proposed in [20] highlights the impact of beam divergence and cross-talk effect between OAM modes. Despite this, the efficient generation and transmission of higher order OAM beam requires challenges in terms of element design and feed network configuration. A four-mode OAM antenna array is proposed in [21], which simultaneously excites multiple modes (0,−1,−2,and −3), each with an equal divergence angle. The design, however, employs four layers of antenna elements, four layers of substrates, and two layers of feeding networks, thereby increasing the overall complexity of the system. Furthermore, the generation of higher-order OAM beams using the proposed technique requires a significant number of antenna elements, which increases the overall size of the array. The uniform circular arrays proposed in [22] and [23] introduce the use of PIN diodes for switching between polarization states and OAM modes, respectively. However, they require a significantly larger array with more antenna elements and precise phase adjustment techniques to support higher-order OAM beam generation effectively.

This paper presents the design of a uniform circular array, in which the OAM mode of the resultant beam is equal to the sum of the OAM modes of array and patch. This approach relaxes the condition of the required number of antenna elements (*N*>2*l*) where *l* is the mode order. In addition, the edge feed mechanism is proposed along with the sequential rotation of elements, thereby reducing the complexity of the feeding mechanism. Sect 1 of this paper presents the analysis and design consideration. Sect 2 represents the results and discussion. Sect 3 represents the experimental validation. The final section concludes the paper.

## 1 Analysis and design consideration

### 1.1 Analytical model

**Fig 1:**
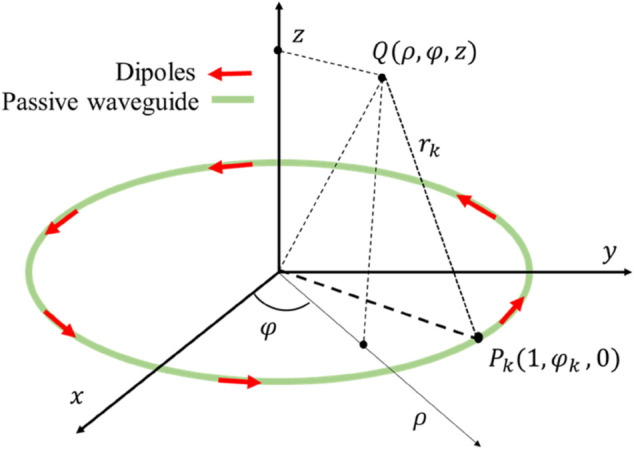
Analytical model of UCA.

Fig 1 represents the analytical model of the uniform circular array in the cylindrical coordinate system. Consider k = 1,2,3,...q dipoles are distributed equally along the circumference with ρ=1 along the plane (z = 0).

The analytical model is analyzed in the cylindrical coordinate (*ρ*,φ,z), where z is the normalized distance of the resonator with radius D from the ring plane and *ρ* is the polar radius. The azimuthal dipole *P*_*k*_ is related to the mode order *l* as follows:

$$P_{k}=\varphi_{k}P_{o}e^{jl\varphi_{k}}$$ (1)

In the above expression, *P*_*o*_ represents the uniform dipole moment, $\varphi_{k}$ represents the azimuthal angle of k-th dipole. The electric field obtained from the interference of all dipoles radiations at point Q(*ρ*,φ,z) is given as [24]:

$$𝐄(\rho ,\varphi ,z)=\frac{B}{D^{3}}\sum_{k=1}^{q}\exp(jvd_{k})\exp(jl\phi_{k})\;\;\times \left(\frac{v^{2}}{d_{k}}-j\frac{v}{d_{k}^{2}}-\frac{1}{d_{k}^{3}}\right)(\hat{d_{k}}\times \hat{\varphi_{k}})\times \hat{d_{k}}\;-\left(j\frac{2v}{d_{k}^{2}}+\frac{2}{d_{k}^{3}}\right)(\hat{d_{k}}\cdot \hat{\varphi_{k}})\hat{d_{k}}$$ (2)

where B is the constant which depends on the permittivity of the medium and uniform dipole *P*_*o*_, *d*_*k*_ is the distance of kth dipole point *P*_*k*_ from the point of observation Q, $\hat{d_{k}}$ represents the direction of *P*_*k*_*Q*, originating from *P*_*k*_.

### 1.2 Modal analysis and modes superposition

**Fig 2:**
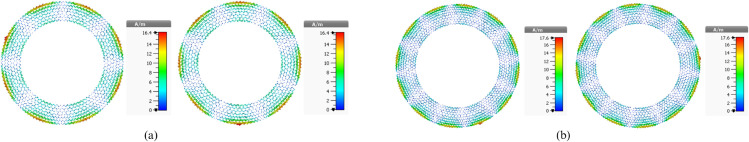
Characteristic current distribution at 3.55 GHz. (a) *TM*_41_ degenerate modes (b) *TM*_61_ degenerate modes.

Characteristic Mode Analysis (CMA) is performed to observe the excitation of *TM*_*nm*_ modes required for generating a specific-order orbital angular momentum (OAM) mode. According to characteristic mode theory, when two degenerate *TM*_*nm*_ modes are excited with equal amplitude and a 90-degree phase difference, they can radiate an OAM wave of mode order (*n*–1) [25]. Fig 2 represents the current distribution of the degenerate mode pair analyzed using CST STUDIO at 3.55 GHz. A ring configuration with an inner radius of 27 mm and an outer radius of 40 mm is analyzed through CMA as proposed in [26,27], with a mesh density of $\lambda_{0}/40$, where $\lambda_{0}$ is the free-space wavelength. It can be seen that the degenerate modes observed in Fig 2(a) are from *TM*_41_ mode as each of them has eight alternate maximum points. Hence, they can be excited to form third order OAM beam according to modes theory. Similarly, degenerate modes of Fig 2(b) are from *TM*_61_ mode as each of them has twelve alternate maximum points and can be excited to form fifth order OAM wave. The condition for the number of elements required for a certain mode-order OAM is provided in [28], stating that the number of elements should be greater than twice the topological charge when individual elements carry no OAM. The resultant E-field for the antenna array can be expressed in the cylindrical coordinate system as follows:

$$E_{\text{array}}(\rho ,\varphi ,z)=A(\rho ,\varphi ,z)\psi (\rho ,\varphi ,z)$$ (3)

where A(ρ,φ,z) represents antenna element factor which carry no OAM and ψ(ρ,φ,z) represents array factor. Contrary to antenna elements which do not carry an OAM in [29], array has OAM with phase factor $e^{jl\varphi }$. According to the multiplication theorem, substituting the antenna elements with OAM-carrying elements that possess the spatial factor would modify the array’s total phase factor as follows:

$$e^{jl_{t}\varphi }=e^{jl_{e}\varphi }\times e^{jl_{a}\varphi }\;=e^{j(l_{e}+l_{a})\varphi }$$ (4)

where *l*_*t*_ represents the total angular momentum, *l*_*e*_ is the angular momentum of the antenna element and *l*_*a*_ is the angular momentum of the array. The above equation can be further simplified as follows:

$$l_{t}=l_{e}+l_{a}$$ (5)

From the above relation, it is clear that the OAM modes of the array and antenna element can be combined. Based on this fact, the required number of elements can be reduced for radiating an OAM wave with a certain topological charge.

### 1.3 Proposed design

**Fig 3:**
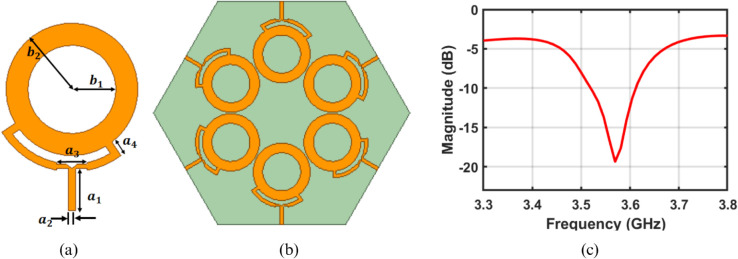
Schematic design of antenna and uniform circular array (a) unit element (b) uniform circular array, (c) impedance characteristic of array, where *b*_1_ = 27*mm*, *b*_2_ = 40*mm*, *a*_1_ = 25*mm*, *a*_2_ = 4.5*mm*, *a*_3_ = 18*mm* and *a*_4_ = 9.5*mm.*

Fig 3(a) illustrates the schematic diagram of the single-ring patch antenna used in the design of the proposed array. The antenna is fabricated on an FR4 substrate with a relative permittivity of 4.4 and a thickness of 1.6 mm. The single element has a ring-shaped structure with inner and outer radii denoted by *b*_1_ and *b*_2_, respectively. The proposed ring patch employs a dual-feed configuration, allowing excitation from two points with a 90-degree phase difference and an angular spacing of 90 degrees. The required phase difference is achieved by introducing a difference in the electrical lengths of the two feed lines, thereby fulfilling the condition for circular polarization. The unit element is designed to radiate a fifth-order circular polarized OAM beam. These elements are arranged along the circumference, with each element equidistant from the center, to form a uniform circular array, as observed in Fig 3(b). The ground and substrate are hexagonal to provide the edge feed configuration. The required phase difference between the adjacent elements of antenna is calculated using the following equation [28].

$$\Delta \phi =\frac{2\pi \ell }{n}$$ (6)

where Δϕ is the phase difference between adjacent elements, ℓ is the mode order and *n* are the number of elements. In the proposed design, the antenna elements are sequentially rotated by $60^{\circ }$, corresponding to an array of *n* = 8 elements and an array OAM mode order of ℓ=1. As a result, the first mode of array is combined with the fifth mode of antenna element to generate the resultant sixth order circular polarized OAM wave.

## 2 Results and discussion

### 2.1 Impedance characteristics

Fig 3(c) illustrates the impedance characteristics of the proposed antenna array. The results indicate that the antenna operates in the frequency range of 3.51 GHz to 3.60 GHz, with an S-parameter of less than -10 dB. This characteristic makes it well-suited for compact wireless sensor systems and IoT systems operating in the sub-6 GHz range [30–32].

### 2.2 Near field characteristics

**Fig 4:**
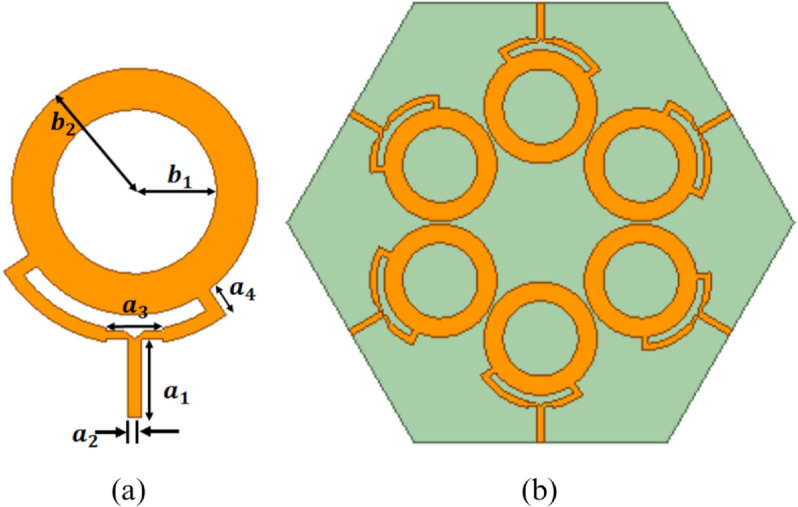
Simulated phase and amplitude distribution of the proposed antenna (a) simulated phase and amplitude for element, (b) resultant simulated phase and amplitude for array.

Fig 4(a) and 4(b) represent the total E-field phase and amplitude distribution of the antenna element and array, respectively. Since the dipoles are replaced with the OAM carrying elements, the resultant field of the array is equal to the sum of the OAM of the element and array. The element in this array has an OAM field with fifth mode order *l*=5. The fifth-order OAM field of the element is combined with the first-order OAM field of the array to generate the resultant OAM field of the sixth order. This feature overcomes the restriction of the required number of elements N for a given mode order *l* i.e. *N*>2*l*. Consequently, the structure becomes less complex due to a decrease in the number of elements for a specific order OAM beam.

### 2.3 Far field characteristics

**Fig 5:**
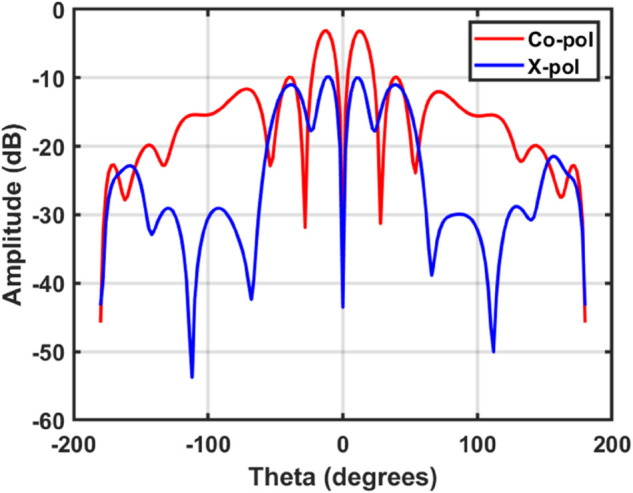
Simulated co-polarization and cross-polarization radiation patterns of the proposed antenna.

Fig 5 represents the simulated co- and cross-polarization radiation patterns of the proposed antenna. It can be observed that the co-polarized component (LHCP) dominates in the main radiation region, while the cross-polarized component (RHCP) remains significantly lower, thereby confirming the polarization performance. Although the cross-polarization level increases at certain off-axis angles due to the presence of sidelobes and nulls, this is a common characteristic of higher-order OAM beams and does not affect the primary polarization performance.

### 2.4 Polarization characteristics

**Fig 6:**
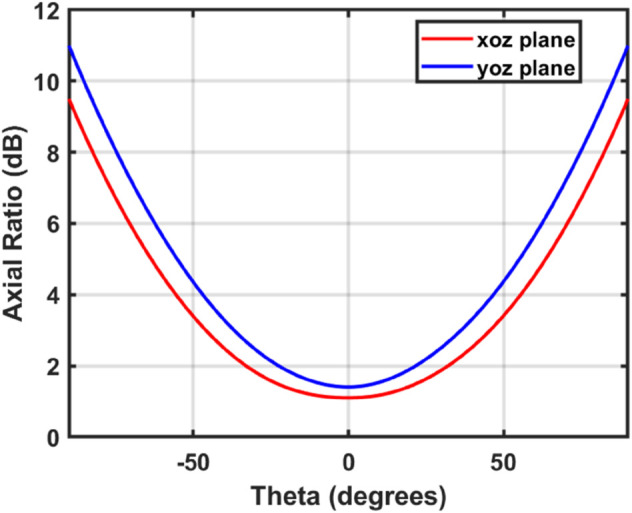
Simulated axial ratio of the proposed antenna at both orthogonal planes.

Fig 6 represents the variation of the axial ratio with the elevation angle (*θ*) at 3.55 GHz for the two main planes.i.e. $\phi =0^{\circ }$ (xoz plane) and $\phi =90^{\circ }$ (yoz plane). The axial ratio stays below 3 dB within $\pm 30^{\circ }$ of the boresight in both planes, which shows that the main radiation area has stable circular polarization. The small difference between the two cuts is due to the fact that the array geometry and feeding structure are not symmetrical, which is common in compact OAM antenna arrays. The main reason for the higher axial ratio at larger off-axis angles is sidelobe effects which does not affect the performance of the main beam’s polarization.

### 2.5 Mode purity

**Fig 7:**
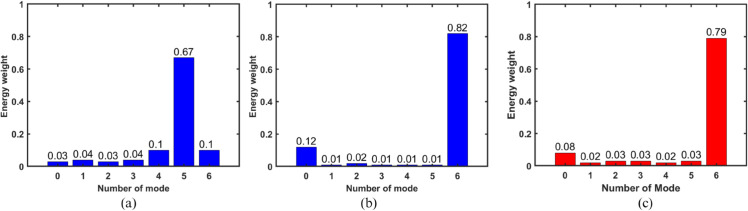
Mode purity (a) for element (simulated) (b) for array (simulated) (c) for array (measured).

The realization of the required order OAM beam can be analyzed through the mode concentration to their topological charges. Fig 7 represents the mode purity of the proposed antenna array. It can be observed that the antenna element exhibits a mode purity of 67 percent for the fifth-order OAM wave. Furthermore, the proposed uniform circular array (UCA) achieves a mode purity of 82 percent for the sixth-order OAM beam, which is the resultant mode order of the patch antenna and UCA.

## 3 Experimental validation

### 3.1 Measurement setup

**Fig 8:**
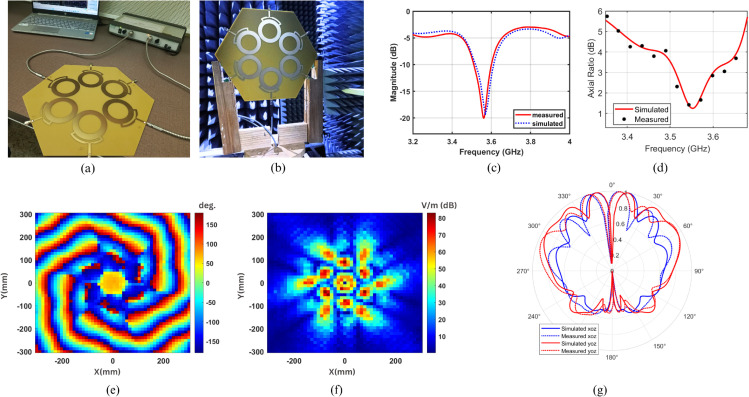
Fabricated Prototype and measurements (a) s-parameter measurement setup (b) far field setup (c) reflection coefficient (d) axial ratio (e) E-field phase (f) E-field amplitude (g) far field pattern.

Fig 8(a) represents the fabricated prototype along with the physical setup used to measure its impedance characteristics. The proposed antenna is connected to a Vector Network Analyzer (VNA), which transmits signals over a specified frequency range and measures the amount of signal reflected back. This helps verify that the antenna resonates within the desired frequency range. Fig 8(b) illustrates the far-field measurement setup, where the antenna is mounted vertically inside an anechoic chamber to evaluate its radiation patterns. The chamber is lined with pyramid-shaped absorbers to eliminate unwanted electromagnetic (EM) reflections. The antenna is positioned on a stand, facing the measurement probe placed on the opposite side.

### 3.2 Comparison of simulated vs. measured results

Fig 8(c) represents the simulated and measured reflection coefficient of the proposed antenna array. It can be observed that the antenna resonates at a frequency of 3.55 GHz, with active S-parameter values less than –10 dB. The simulated and measured results match well, thereby verifying the antenna performance. Fig 8(d) represents the simulated and measured polarization characteristics of the proposed antenna array. It can be observed that the proposed UCA exhibits the axial ratio value of less than 3 dB in the frequency range of 3.55 GHz. This confirms the excitation of circular polarized radiation in the corresponding frequency range. The simulated and measured results for axial ratio are in good agreement. Fig 8(e) and 8(f) represent the measured E-field phase and amplitude, respectively, plotted at a distance of 50 mm from the antenna plane. The near-field *E*-field amplitude and phase measurements are carried out inside an anechoic chamber using a standard open-ended waveguide probe connected to a Vector Network Analyzer (VNA). The probe is mounted on an automated two-dimensional scanning positioner to record the complex field distribution over a planar surface. The measurement plane is located at a fixed distance of 50 mm from the antenna aperture, corresponding to the region where the near-field pattern clearly exhibits the helical phase structure associated with the OAM mode. The scanned area covers 600 mm × 600 mm with a spatial sampling step of 2 mm in both the *x* and *y* directions. The spatial phase distribution exhibits six twists in the anticlockwise direction, confirming the radiation of a left-hand circularly polarized (LHCP) OAM wave. The E-field amplitude also displays six distinct peak points along the edges, further validating the excitation of the desired sixth-order OAM mode. Fig 8(g) represents the simulated and measured far-field pattern of the proposed antenna array along both orthogonal planes, namely the *xoz* and *yoz* planes. It can be observed that the proposed UCA has null along the direction of propagation, which is the unique characteristic of OAM beam. However, the measured *E*-field amplitude does not exhibit a perfect null at the beam center, as theoretically expected for an ideal OAM mode. This small residual field is attributed to several practical factors, including the finite probe aperture size, minor misalignment between the probe and the antenna axis, limited spatial sampling resolution, and slight amplitude and phase imbalance among the array elements. In addition, coupling between adjacent modes and measurement noise can introduce a weak nonzero field near the vortex core. Nevertheless, the measured phase distribution maintains a continuous 2πℓ helical progression, confirming the presence of the OAM mode and validating the overall field characteristics.

The OAM mode purity of the proposed antenna is evaluated using both full-wave simulation and measured near-field data. The complex near-field E(ρ,ϕ) is sampled on a plane at z=50 mm for both HFSS simulations and experimental measurements. The OAM spectrum is obtained through azimuthal Fourier decomposition as follows

$${\hat{c}}_{\ell }(\rho_{0})=\frac{1}{N}\sum_{k=1}^{N}E(\rho_{0},\phi_{k})e^{-j\ell \phi_{k}},$$ (7)

where ℓ represents the mode order and *N* is the total number of sampling points along the azimuth. The mode purity for a particular order ℓ is calculated using

$$\eta_{\ell }=\frac{|{\hat{c}}_{\ell }{|}^{2}}{\sum_{m}|{\hat{c}}_{m}{|}^{2}}.$$ (8)

The sensitivity for radius selection is reduced by taking the average of $\eta_{\ell }$ over a narrow radial annulus centered around the dominant intensity ring. Identical post-processing, including field normalization and phase unwrapping, is applied to both simulated and measured data to ensure a consistent comparison. Fig 7(a)–7(b) represent the mode purity of the proposed antenna element and array, respectively. It can be observed that the antenna element exhibits a mode purity of 67 percent for the fifth-order OAM wave. Furthermore, the proposed uniform circular array (UCA) achieves a mode purity of 82 percent for the sixth-order OAM beam, which is the resultant mode order of the patch antenna and UCA. Fig 7(c) represents the mode purity of proposed antenna array from measured near field data. The measured mode purity is slightly lower than the simulated value due to practical factors such as minor probe misalignment, amplitude and phase imbalance, finite sampling resolution, and residual chamber reflections. Nevertheless, the close agreement between the two validates the accuracy and reliability of the proposed antenna design.

Table 1 compares the proposed UCA with the reported ones. It can be observed that the proposed array operates at 3.55 GHz and exhibits 2.5% fractional bandwidth which is similar to or slightly narrower than many previous single-band designs such as [15,16,18,22,23] and is recommended for narrow band applications [33,34]. The proposed array has a smaller area than other reported designs [15,17,20,21], which is good for compact applications. The antenna realizes the required sixth-order OAM beam while using a reduced number of elements (6 elements) through the modes superposition approach. The similar designs reported in the literature either generate lower-order modes [22,23] or require more elements to realize the higher-order OAM beam, thereby increasing the overall space and complexity. The proposed UCA relaxes the general requirement of elements |N|>2ℓ and reduces array complexity for high-order OAM generation. Furthermore, the proposed antenna is a single layered design which does not require multi-layer substrates or such feeding configurations in comparison to several other reported techniques. Consequently, overall complexity of the system is reduced.

**Table 1:** Comparison of the proposed UCA with reported designs.

Ref.	Antenna Size (${\lambda_{0}}^{2}$)	f (GHz)	BW (%)	Higher Mode	CP	Elements	Feed Complexity	Multi-layered
[15]	π×2.43×2.43	5.83	3.9	1	No	8	High	Yes
[16]	π×1.23×1.23	9.2	3.6	2	Yes	12	Low	No
[17]	24×24	2.4	3.9	2	No	16	High	Yes
[18]	2.3×2.3	2.4	2.0	2	Yes	4	High	Yes
[19]	2.8×2.8	5.2	7.6	2	No	12	High	Yes
[20]	π×3×3	10	40.0	3	No	8	High	No
[21]	7.8×7.8	5.8	8.6	3	No	16	High	Yes
[22]	2.7×2.7	3.28	3.0	2	Yes	12	Medium	No
[23]	2.25×2.20	2.54	12.5	1	Yes	16	Medium	Yes
This Work	π×2.0×2.0	**3.55**	**2.5**	**6**	**Yes**	**6**	**Low**	**No**

**Note:** CP = Circular Polarization; BW = Bandwidth.

## 4 Conclusion

A compact uniform circular array antenna has been designed to generate a higher-order OAM beam by combining the fifth-order OAM mode of individual elements with the first-order OAM mode of the array. The use of a dual-feed configuration and sequential rotation simplifies the design while maintaining circular polarization. The antenna operates at 3.55 GHz with high mode purity and low feed complexity, making it suitable for narrowband wireless sensor networks and IoT systems.
